# Supplementary material for: PiSpy: An affordable, accessible, and flexible imaging platform for the automated observation of organismal biology and behavior
Source: PLoS One. 2022 Oct 26;17(10):e0276652. doi: 10.1371/journal.pone.0276652 (PMC9604989; doi:10.1371/journal.pone.0276652)
Supplement: S1 File — For assembly and setup instructions, refer to the User’s Manual (also found at (https://github.com/gpask/PiSpy). (PDF) [file pone.0276652.s001.pdf]

PiSPY

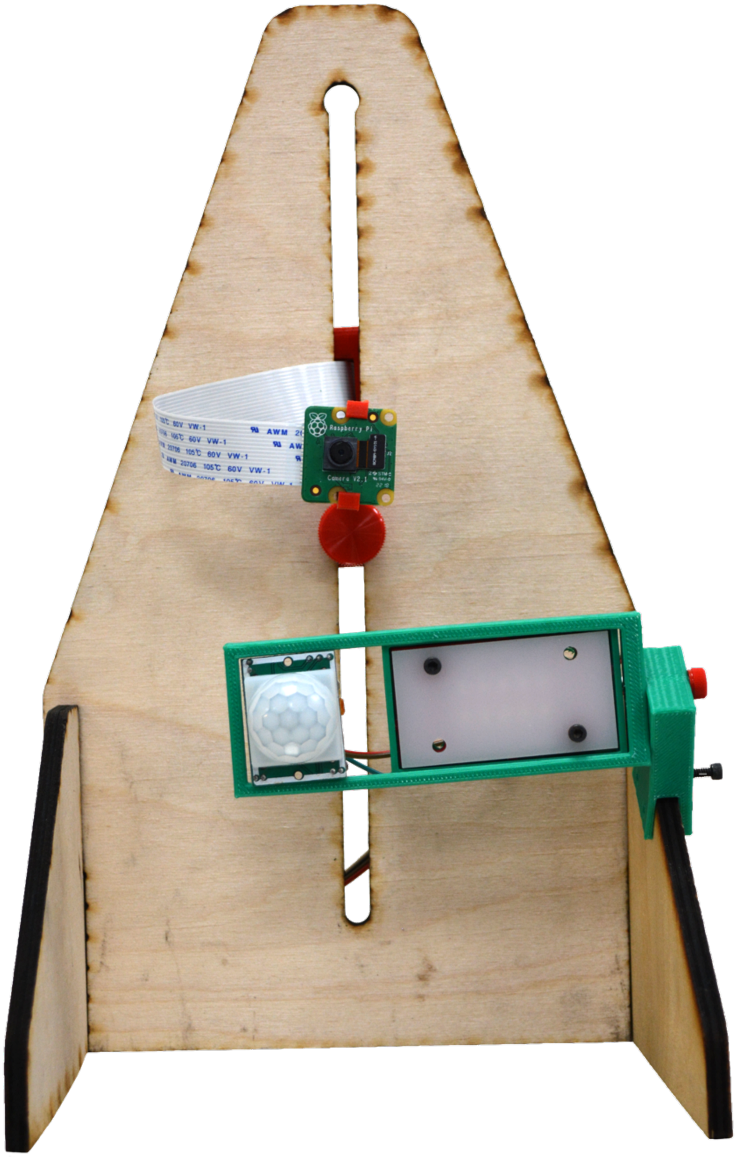

The PiSpy is an affordable device used to automate the acquisition of images and videos. It was shared on bioRxiv in 2022 (https://www.biorxiv.org/content/10.1101/2022.03.21.485129v1). It uses a Raspberry Pi computer and camera to record video and images at specified time intervals or when triggered by an input signal, such as a motion sensor or force plate. Additionally, it can automate the control of LED lights. All settings for the PiSpy are controlled through a graphical user interface that uses the TKinter Python package. The following user guide will outline how to set up and operate the PiSpy. Our testing was with a Raspberry Pi3b or Pi4, which is primarily what this guide is written for, but with some modifications the PiSpy can also be used with older Raspberry Pi models.

Table of Contents

PiSpy Hardware Assembly **3**

Fabrication of Components *3*

Assembly Instructions *3*

Setting up the Raspberry Pi Computer **7**

Installing Raspberry Pi OS *7*

Operating the graphical user interface **12**

Input trigger *14*

Light Control *14*

Note on Red Lights at Night *15*

Quick Capture *15*

Preview *15*

File Identifier *15*

Running the PiSpy Program *15*

Connecting GPIO sensors for input trigger **16**

Connecting a PIR motion sensor *16*

Connecting an IR break beam *17*

Note on PIR motion sensors *18*

Note on IR break beams *18*

Note on BOARD vs BCM GPIO labeling *18*

Note on code for input triggers *18*

Using input trigger mode *19*

Optional: connecting a Real-Time Clock (RTC) to the PiSpy *21*

Adjusting Default PiSpy Settings **21**

LED PCB Lighting **24**

PiSpy Hardware Assembly

Fabrication of Components

The hardware files associated with PiSpy can be downloaded from the GitHub repository (https://github.com/gpask/PiSpy/tree/master/Hardware/Original%20PiSpy).

The wooden stand used to mount PiSpy can be laser cut from 3/8 inch plywood using the .svg file. The 3D-printed components were printed with polylactic acid (PLA) using the .stl files.

Assembly Instructions

All the components for assembly are shown below:

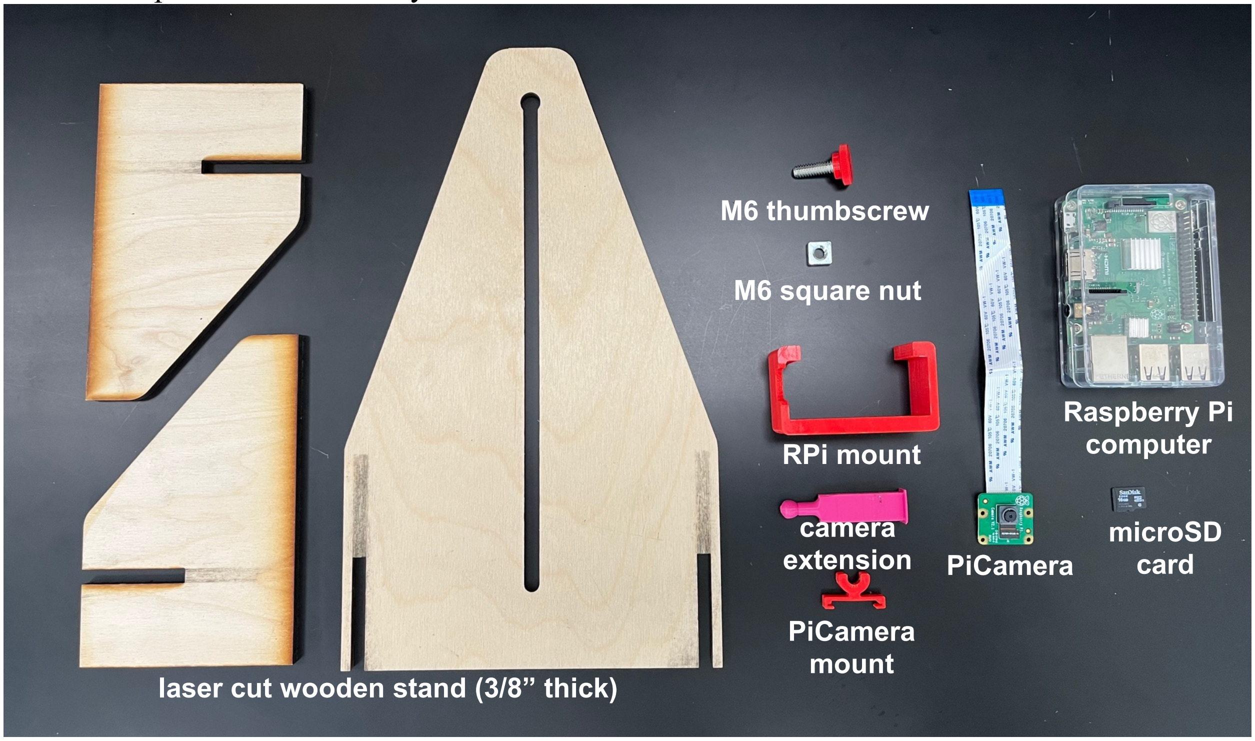

First, assemble the wooden stand by sliding each support into the taller mount like so:

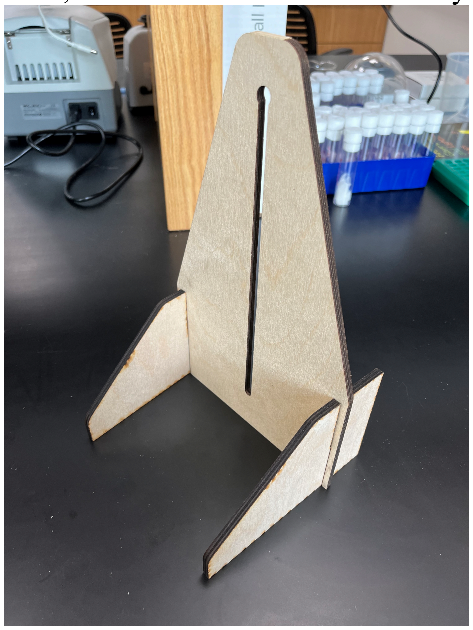

Next, detach the ribbon cable from the PiCamera and insert one end into the Raspberry Pi's camera port.

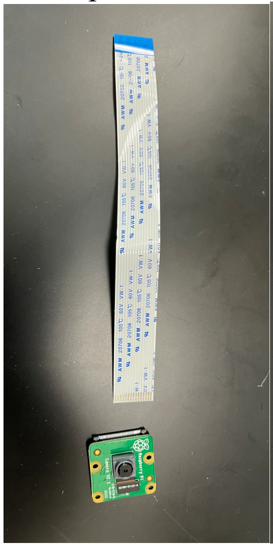

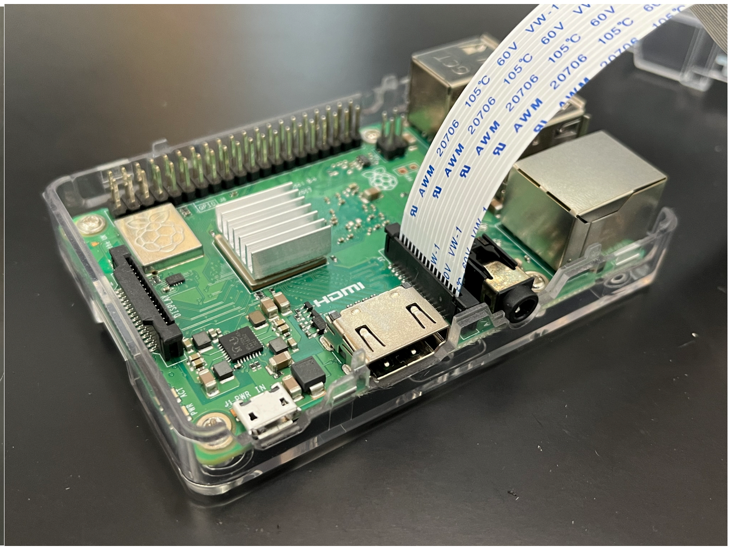

.

Then insert the PiCamera into the PiCamera mount (be sure not to block the ribbon cable connector).

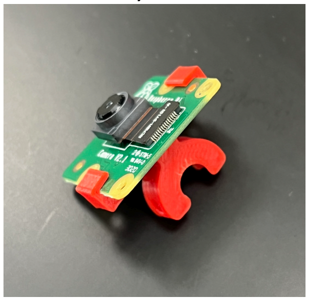

Insert the rounded end of the camera extension through the middle opening of the RPi mount until it is flush with the inner surface.

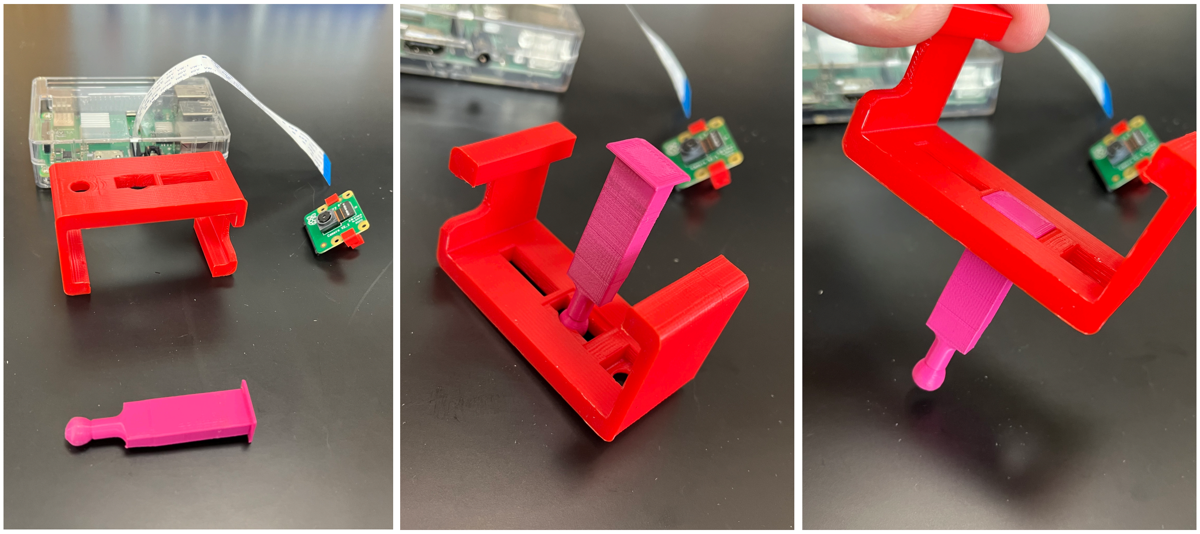

Next, insert the M6 square nut into the RPi mount. Then push the camera extension through the top hole of the wooden mount. Use the M6 thumbscrew to attach the RPi mount to the stand, but do not fully tighten it. The end of the thumbscrew should be flush with the square nut to allow the Raspberry Pi to slide into the RPi mount in the next step.

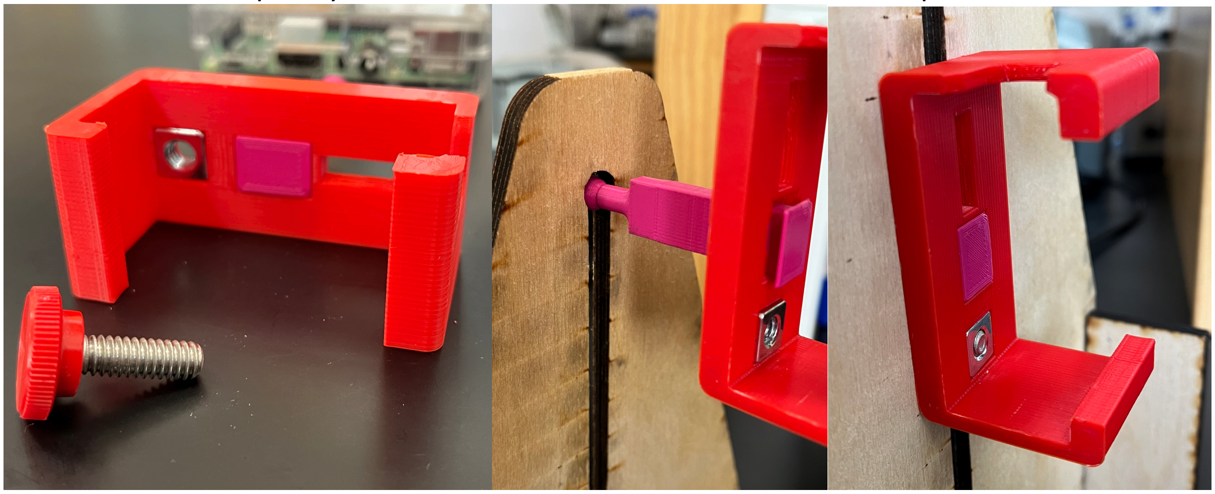

First insert the camera ribbon cable through the top rectangular opening in the RPi mount. Then slide the Raspberry Pi and case into the RPi mount. *(Note: If your specific case does not fit inside RPi mount, you can adjust the scale of that specific axis before 3D printing.)*

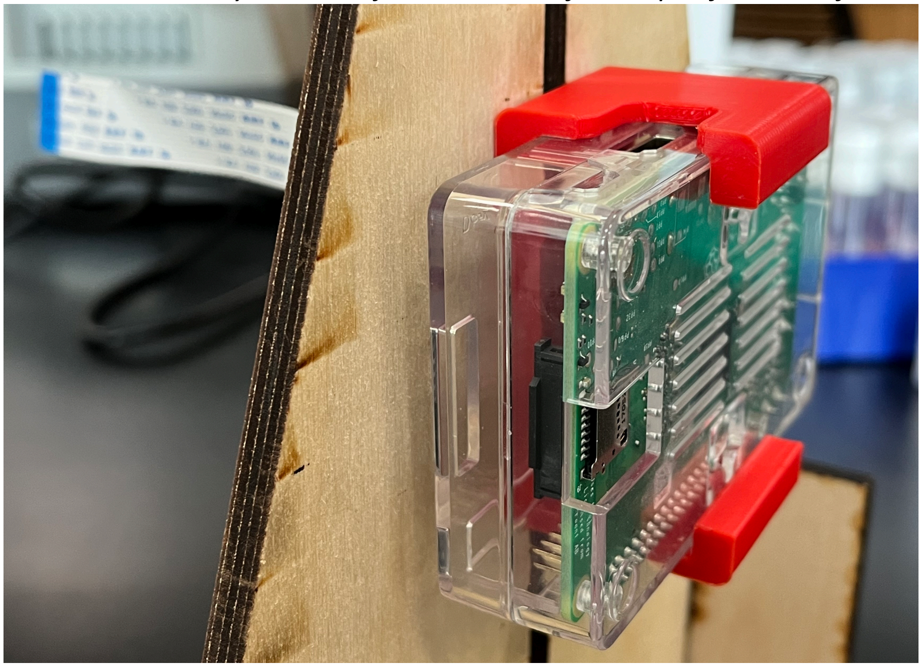

Attach the PiCamera and mount to the “ball” end of the camera extension and connect the ribbon cable.

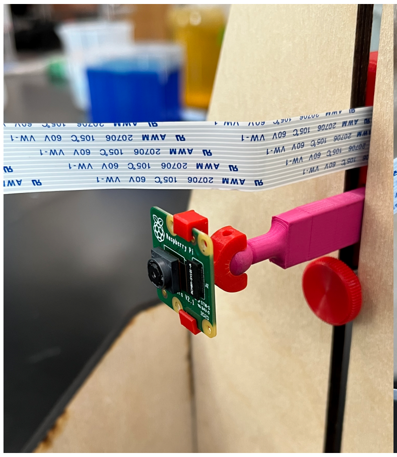
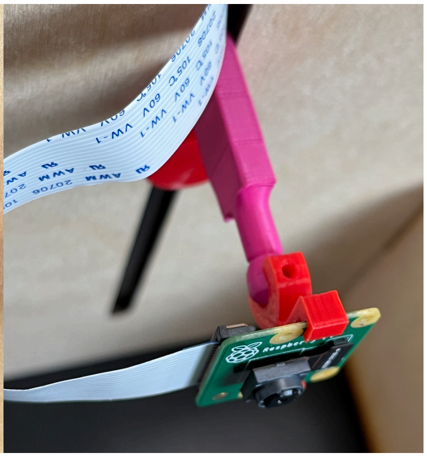

Connect the power supply, HDMI cable, keyboard, and mouse. The microSD card will be used in the next section to load the PiSpy disk image and will be inserted as shown below:

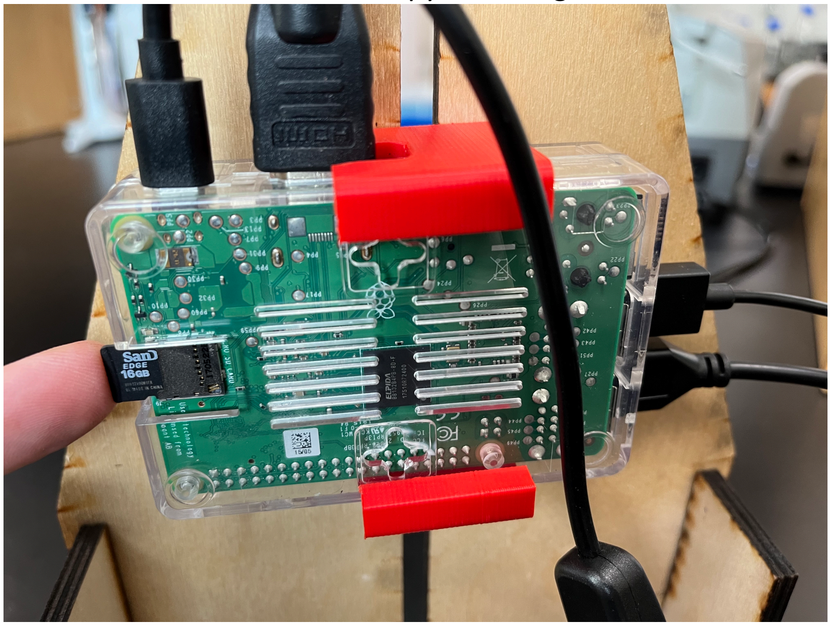

Setting up the Raspberry Pi Computer

Installing Raspberry Pi OS

The first step to running PiSpy is to set up the Raspberry Pi OS operating system on the computer. To do so, on a computer with micro SD card reader, download the Raspberry Pi imager (https://www.raspberrypi.com/software/) and the PiSpy disk image from the GitHub repository (https://github.com/gpask/PiSpy/blob/master/Software/PiSpy%20disk%20image.md). Insert the micro SD card into the computer and open the imager.

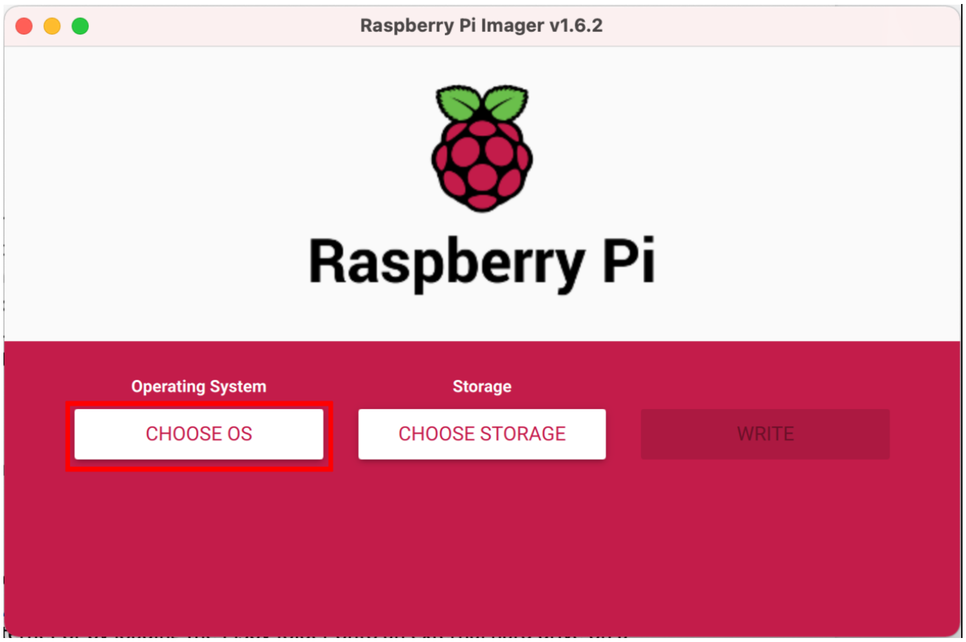

Select "CHOOSE OS,"

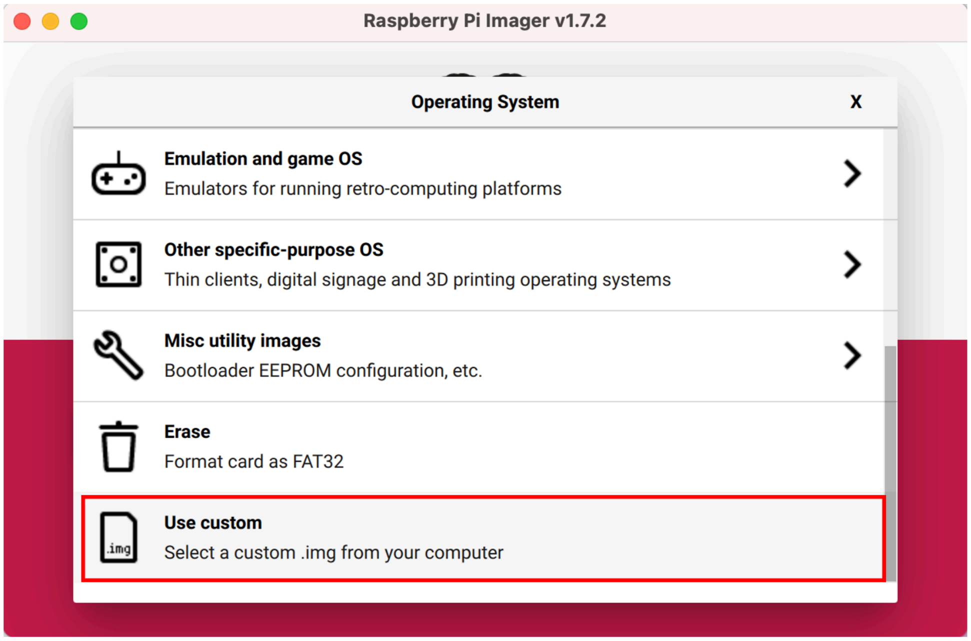

then select "Use custom," and then select the "PiSpy.img" file you just downloaded.

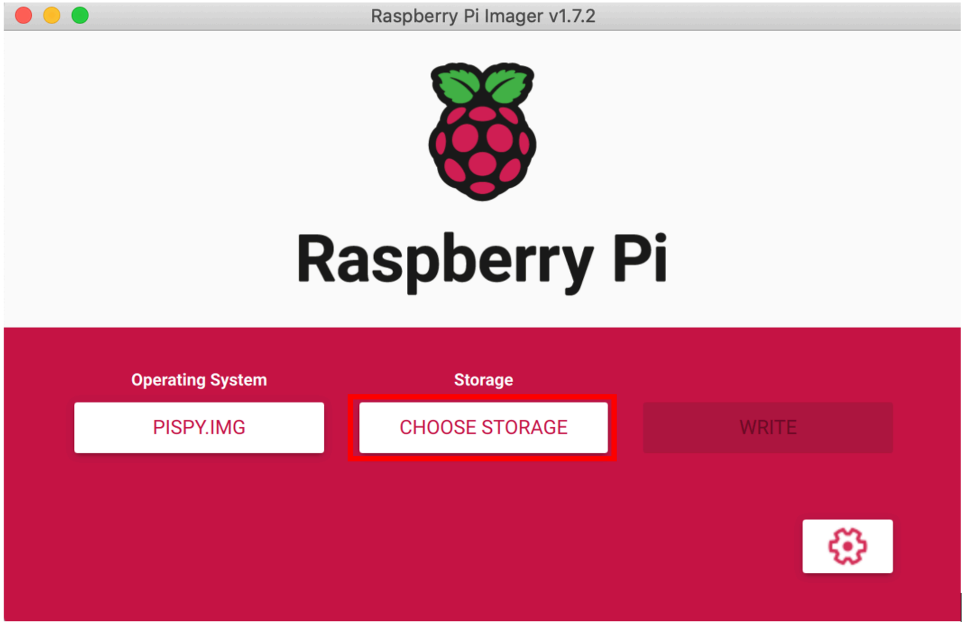

Next, select "CHOOSE STORAGE"

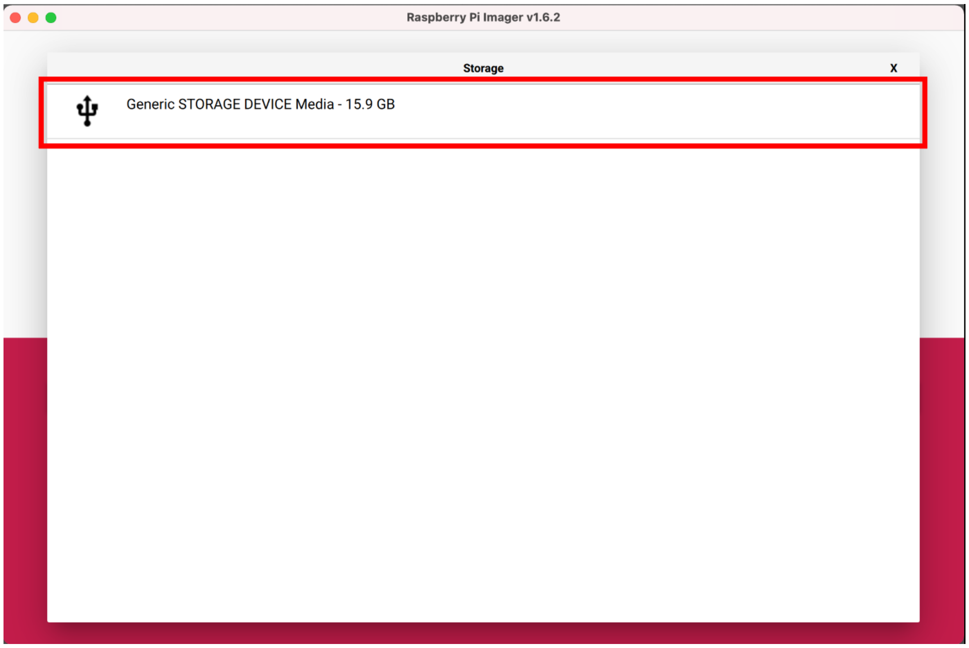

and select the micro SD card to be used for your PiSpy.

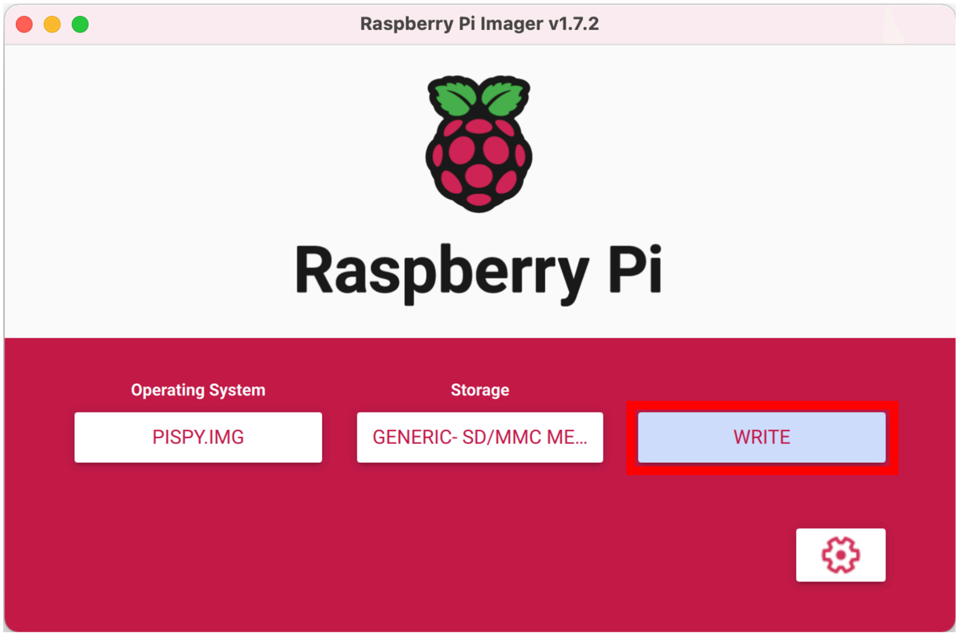

Finally, write the OS to the SD card by selecting "WRITE"

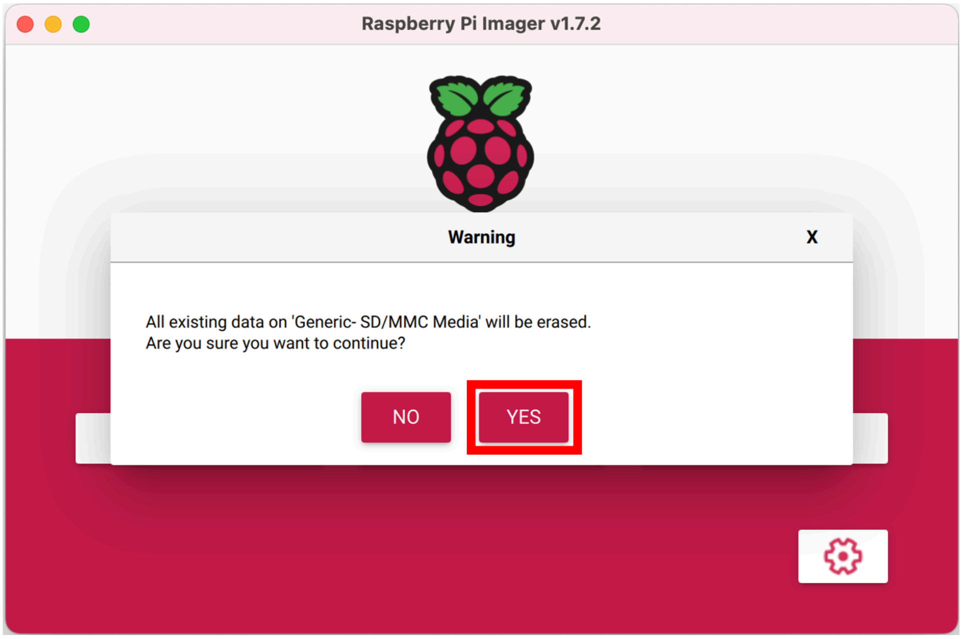

and confirming that this will erase any previous data on the SD card.

When complete, insert the micro SD card into the Raspberry Pi (as shown on page 7) and power it on. Once booted, your desktop should look like this:

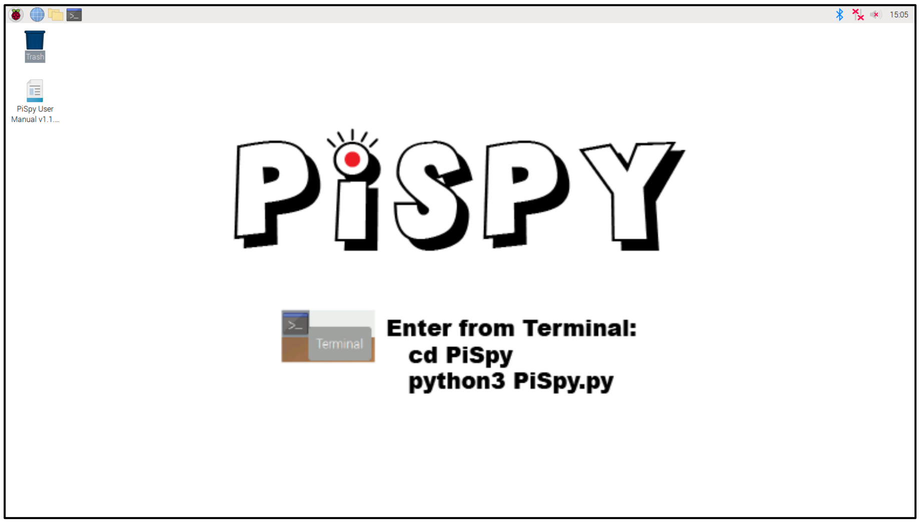

Navigate to the File Manager in the top left corner. You'll notice the folder containing the PiSpy code in the /home/pi folder.

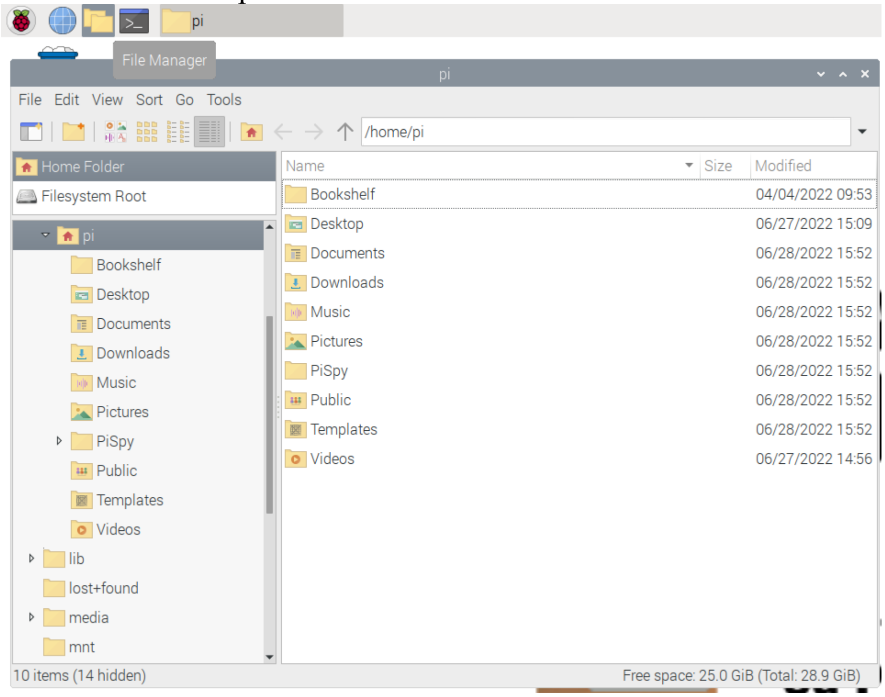

Next, open up Terminal in the top left corner and enter:

```
sudo raspi-config
```

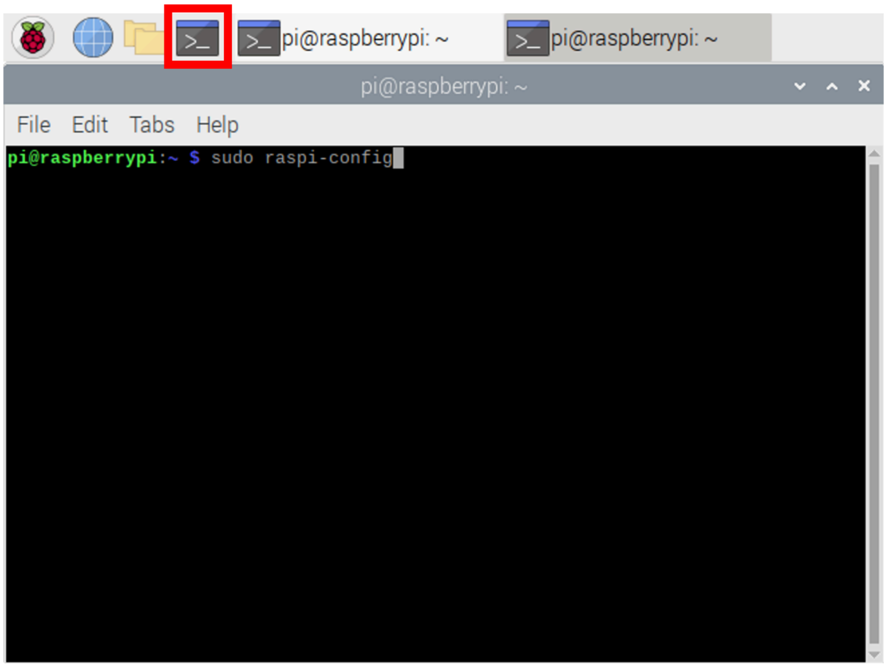

This will open the following menu. Navigate toward "6 Advanced Options" and press the enter key. Then select "A1 Expand Filesystem" and press enter again. This will expand the accessible filesystem to the size of your micro SD card.

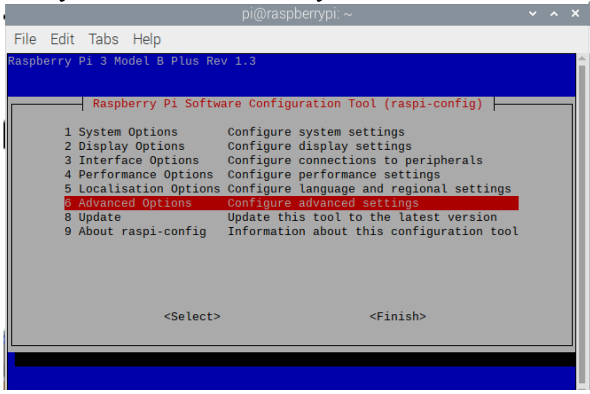

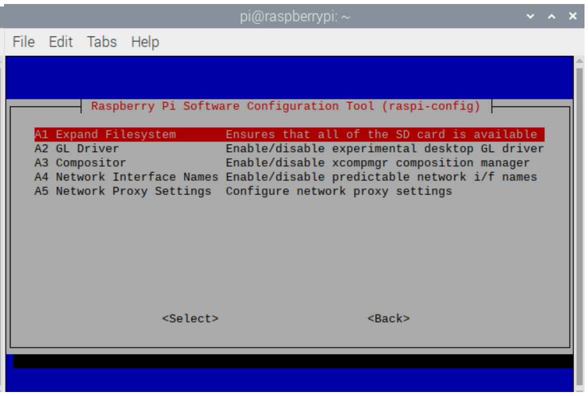

Navigate to "Finish" by pressing the right arrow button, and then select yes to reboot and start filesystem expansion.

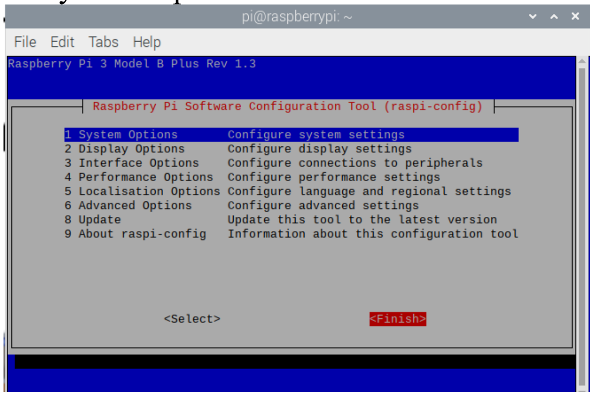

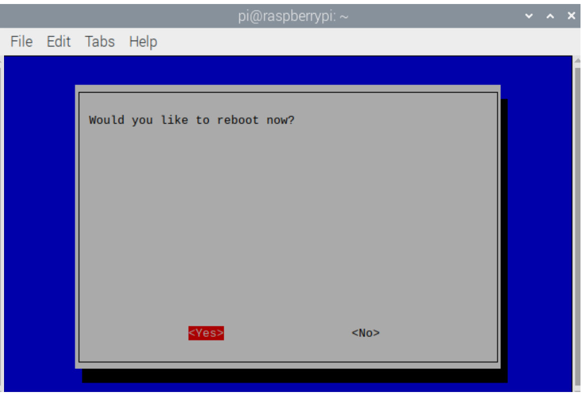

Operating the graphical user interface

To access the GUI, open the command line by clicking on the terminal icon on the top bar of the screen. Then, enter the following commands, first to move to the PiSpy folder and then to open the PiSpy Control window, which is where the PiSpy is operated from:

```
cd PiSpy
python3 PiSpy.py
```

If you have already entered those commands, you can use the up arrow to access previously used commands in terminal. Also note that the `cd PiSpy` will only work if the folder containing the PiSpy code is still named "PiSpy." If the name is changed for some reason (as can happen when unzipping the file), either change the name back to PiSpy or enter the new name for the folder when typing that command.

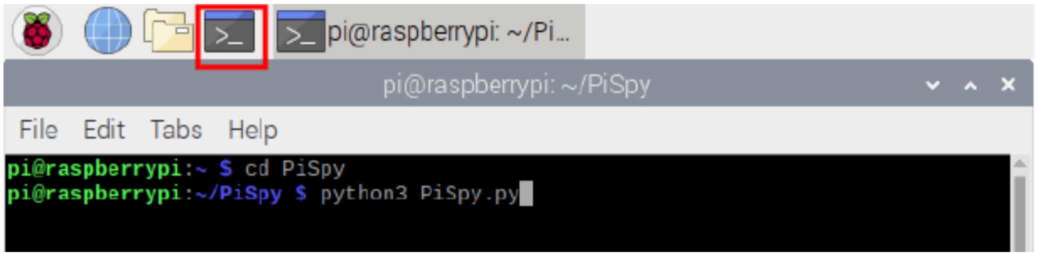

To help remember how to access the GUI, we have included on the Github repository a suggested background for the PiSpy (“PiSpy desktop.png”) and included it in the disk image.

Once the GUI is opened, choose your desired capture mode (image or video) and frequency (timed or triggered) by checking the corresponding boxes. Only one mode and frequency combination (i.e. timed videos) should be selected at a time. Additionally, select the camera resolution by checking the box of the desired resolution, and, for video recording, specify the frame rate. If no resolution is selected, the PiSpy will by default record with a resolution of 1920 x 1080. This link (https://picamera.readthedocs.io/en/release-1.12/fov.html#camera-modes) lists the possible resolutions and framerates for the V1 and V2 PiCameras. Depending on the videos being recorded and the quality of the microSD card used, selecting frame rate/resolution combinations at the upper limits of the accepted range can result in skipped frames. If this occurs, lower either the resolution or the frame rate.

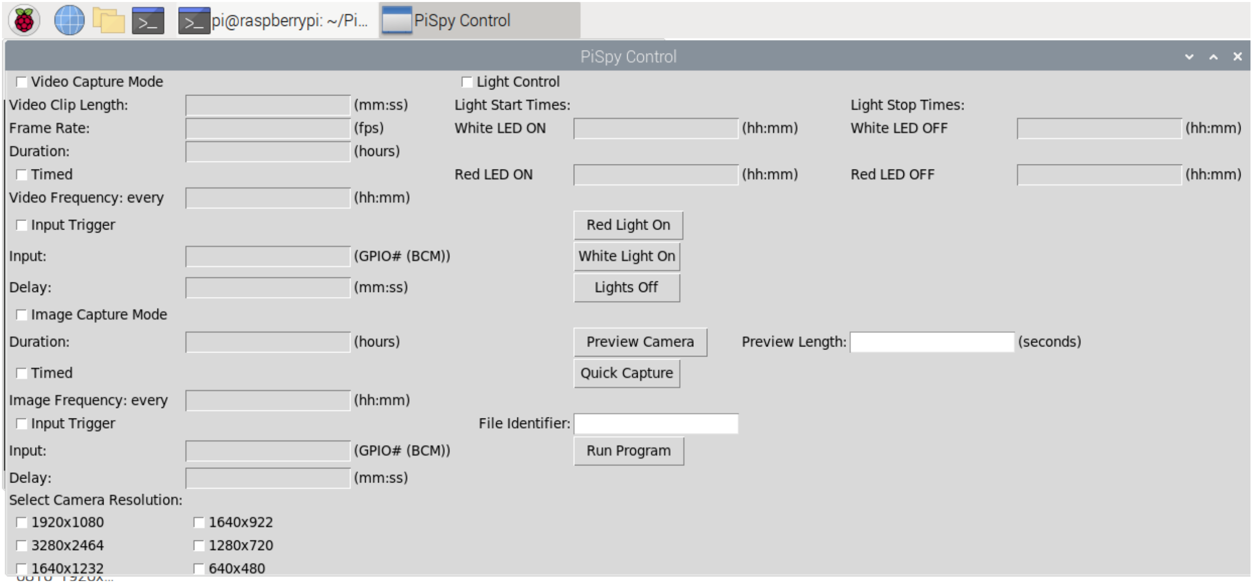

Timed recording:

Timed recording can be used when the user wants images or videos to be recorded at fixed intervals. The image or video frequency should be input in the hh:mm format (using a 24 hour clock), and specifies how often images/videos are recorded. For instance, in the below image, 30 second videos with a frame rate of 20 fps and a resolution of 1920x1080 will be recorded every 1.5 hours for 3 days. Note that the frequency must be less than 24 hours.

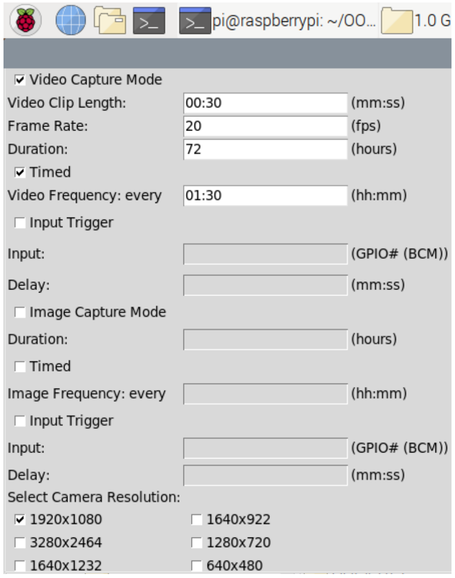

Input trigger

Input trigger mode can be used when the user wants an external source such as a motion sensor or force plate to determine when images or videos are recorded. See **Connecting GPIO sensors for input trigger** for more information on how to use different sensors for input trigger mode.

Light Control

If light control is desired, check the corresponding box and enter the times when each light should be turned on/off (hh:mm format, using a 24-hour clock). Using the red and/or white light buttons, turn on the light(s) you want on when the program starts running. In the image below, the white lights will be on from 7 AM to 7 PM, and the red lights will be on from 7 PM to 7 AM.

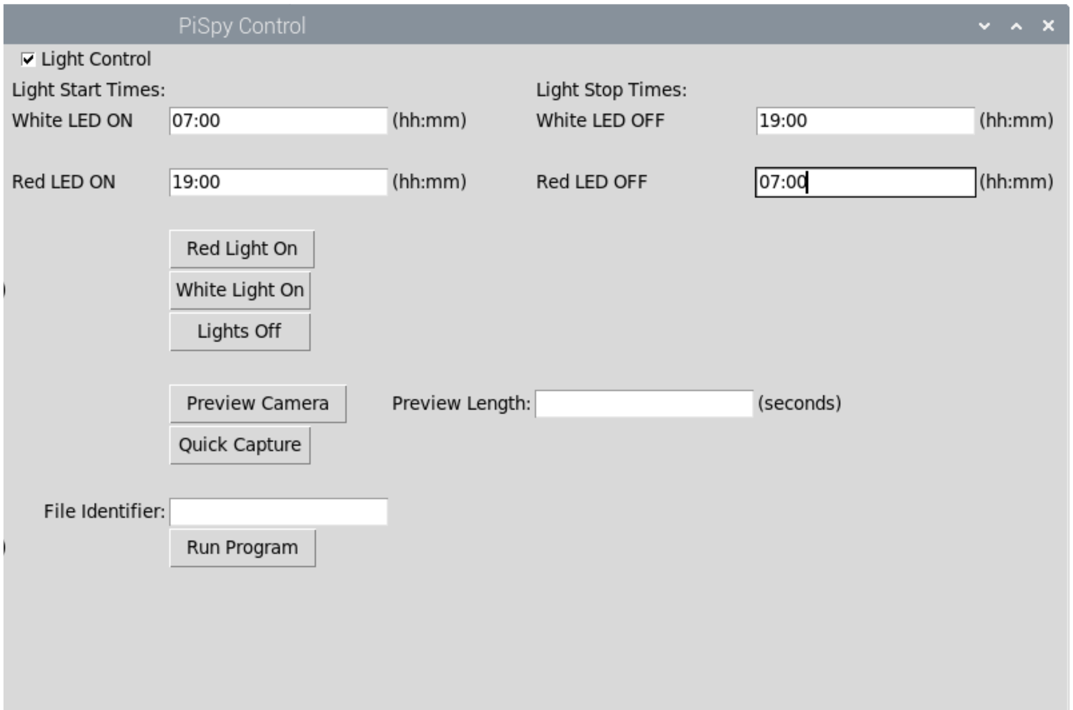

Note on Red Lights at Night
The default settings programmed into the PiSpy are what we found in testing to give the best quality night images/recordings. However, because of the PiCamera's auto white balance settings, if the subject isn't getting enough light or is sending back too much glare towards the camera, images/videos recorded at night can appear washed out. In addition to making changes to the PiSpy settings (see **Adjusting Default PiSpy Settings** below), changing the location and angle of the lights on the PiSpy, the proximity of the subject to the lens, and the height of the camera can all also help with improving night image quality.,

Quick Capture
If the user wants to manually take a picture or video, they can use the quick capture button. Select either video capture mode or image capture mode, and if taking a video also specify the clip length. Then, click the quick capture button to record.

Preview
To preview the output of the camera, the user can select the "preview'' button. The preview length box allows the user to select how long the preview will last for (while the preview window is open, the user cannot interact with the PiSpy Control window). If no time is selected, the PiSpy will default to a 10 second preview.

File Identifier
Optionally, a file identifier can be entered to distinguish between different experiments and/or PiSpy devices. The input in this box will be placed before the default title (local timestamp), but an input here is not required.

Running the PiSpy Program
Once all the settings have been chosen, click the apply button to run the program.
Once the settings are applied, the PiSpy control window will close to run the program, and the chosen settings will be printed out to the terminal window. If the program needs to be stopped before the designated end time, perform a keyboard interrupt in terminal (Control + C) or close the terminal window by clicking the X in the top right corner of the window. This will keep any previously recorded images/videos, but stop the program from recording any more, allowing the user to relaunch the control window and select new settings if desired.

Connecting GPIO sensors for input trigger

Connecting a PIR motion sensor

A PIR motion sensor will have three pins: VCC, GND, and OUT. On some PIR motion sensors, the labels are under the cap; if this is the case, gently remove the cap to be able to identify the pins (the image below is a PIR sensor with the cap removed to show the pin labels). Using the image below as a reference (or by using the "pinout" command in terminal), connect the VCC pin to a 5V or 3.3V power supply pin on the Raspberry Pi, the GND pin to a ground pin on the Pi, and the OUT pin to any of the numbered GPIO pins on the Pi. GPIO board numbering differs between different models of the Raspberry Pi, so if you are not using the Raspberry Pi 3 you will need to consult a different chart when connecting the sensor. The OUT will output a voltage when the sensor is activated, which will be received by the Raspberry Pi and trigger the PiSpy to record an image/video (depending on selected settings). The diagram below is just a suggestion and does not need to be followed exactly; the sensor will work as long as GND is plugged into a ground pin, OUT to a GPIO pin, and VCC to a power supply.

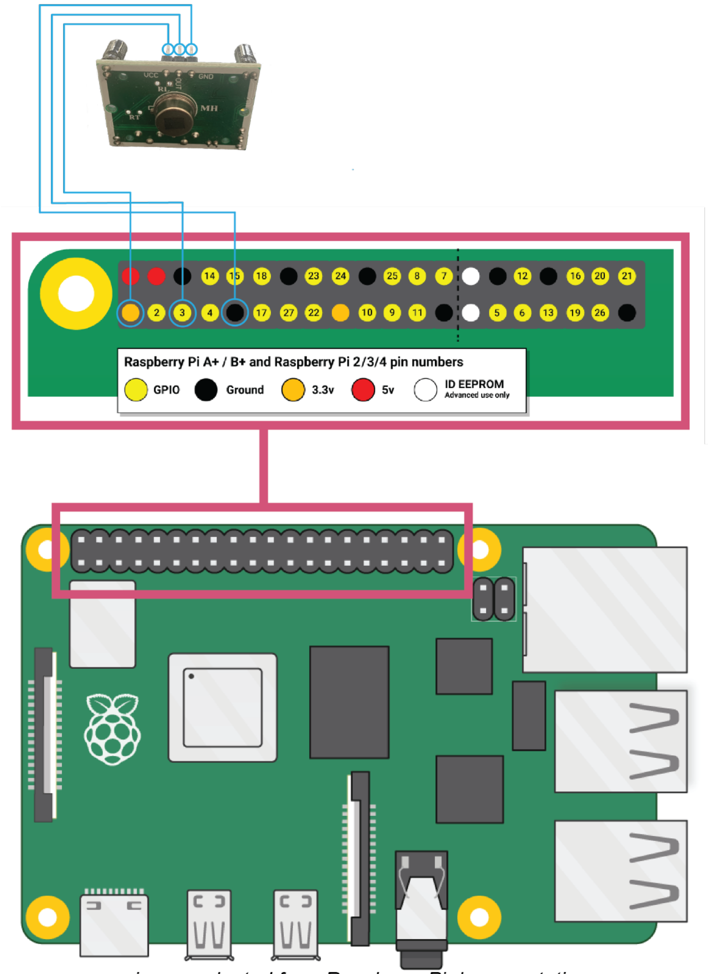

image adapted from Raspberry Pi documentation

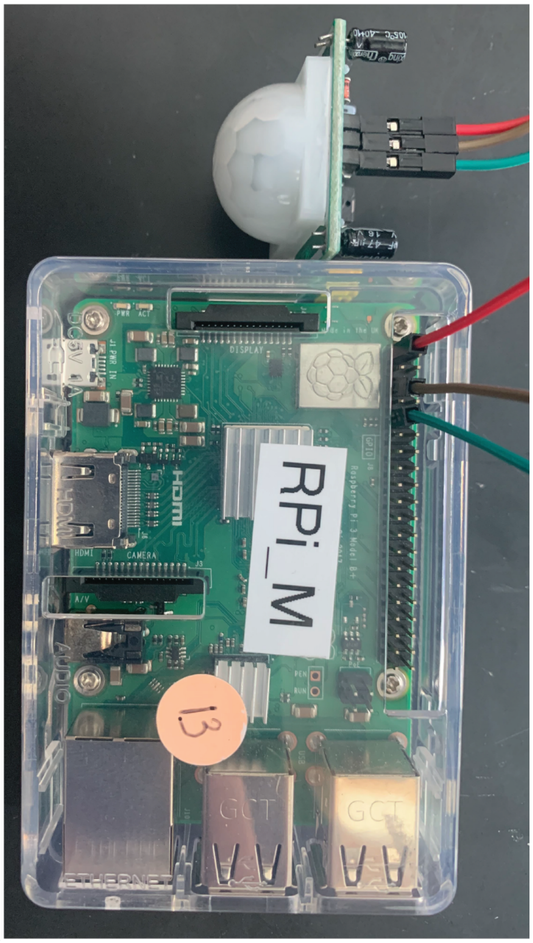

Connecting an IR break beam

An IR break beam has two components, an emitter and a receiver, but the wiring is similar to a motion sensor. The emitter emits a beam of IR light and has two wires, a red one that receives power from a 5V or 3.3V (the range will be better if using 5V) power supply on the Raspberry Pi, and a black one that connects to a ground pin. The receiver, which receives the signal from the emitter, has the same red and black wires which must be plugged into a different power and ground pin, but also has a white or yellow output wire that should be plugged into one of the general purpose GPIO pins. See the image below for reference. As noted above, the power can come from a 3.3V pin, but the range of the break beam will be smaller. Additionally, any GPIO pin can be used, not just 2 (just make sure to specify which one the input is plugged into when you run the PiSpy)

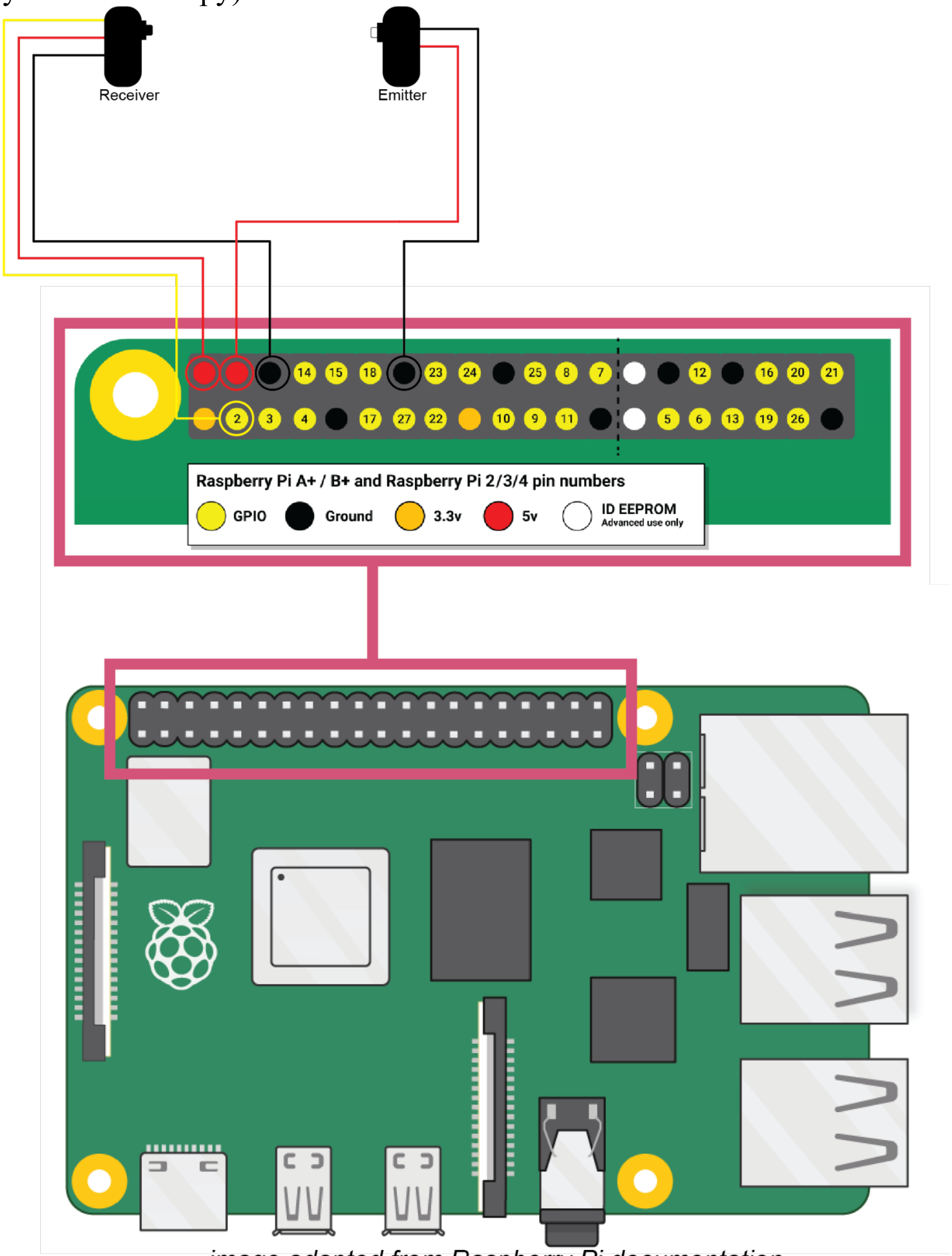

image adapted from Raspberry Pi documentation

Note on PIR motion sensors
In our testing, several commercially available and inexpensive PIR motion simply do not function. If the motion sensor does not correctly trigger image/video acquisition, double check that every connection is in the proper position and secure, and if that doesn't work, consider trying a different sensor.

Note on IR break beams
An IR break beam has two parts, an emitter that sends out IR light and a sensor that receives that signal and will signal to the computer when that beam is broken. In our testing, small objects (we were using ants) don't always reliably break the beam and trigger the sensor. To make the sensor sensitive enough to trigger when an ant passed it, we used LEGOs® to decrease the total area that the sensor could receive IR signal from, and placed a single index card in front of the sensor as well. The index card is partially transparent to IR light, so when nothing else was between the sensor and the emitter the beam was not broken, but when an ant passed it would trigger video acquisition. Our suggestion is to test break beam sensitivity with your desired subject before running any experiment to confirm that the beam will trigger, and if it does not increase the sensitivity, which can be done with DIY tools and further testing as we did.

Note on BOARD vs BCM GPIO labeling
There are two different ways Raspberry Pi GPIO pins can be referred to. BOARD labeling refers to the actual position of the pin on the GPIO board, whereas BCM (Broadcom chip-specific pin numbers) refers to the lower-level functions of the pins. The PiSpy uses the BCM labelling throughout its code, so this is how the input source must be referred to when using input trigger mode.

Note on code for input triggers
Different input triggers function in slightly different ways, and the code (in the "Input_Trigger.py" file) must be slightly different for each of them. As currently deposited on GitHub, the code is written to work with an IR break beam, which has an output of 1 when the beam is not broken and 0 when the beam is broken. As advised in the Input_Trigger.py code, if using a PIR motion sensor, which outputs a 0 when it does not sense motion and a 1 when it does, change lines 56 and 113 of the Input_Trigger.py document from "if x == 0:" to "if i >= 1:" and comment out (place # signs before) lines 50-54 and 108-112. See the screenshots below for reference.

While we do not provide code/instructions for wiring of other types of input triggers besides PIR motion sensors and IR break beams, there are a considerable number of online resources available to help with any other types of sensor, many of which can be found directly on websites like Adafruit and raspberrypi.com when purchasing the sensors.

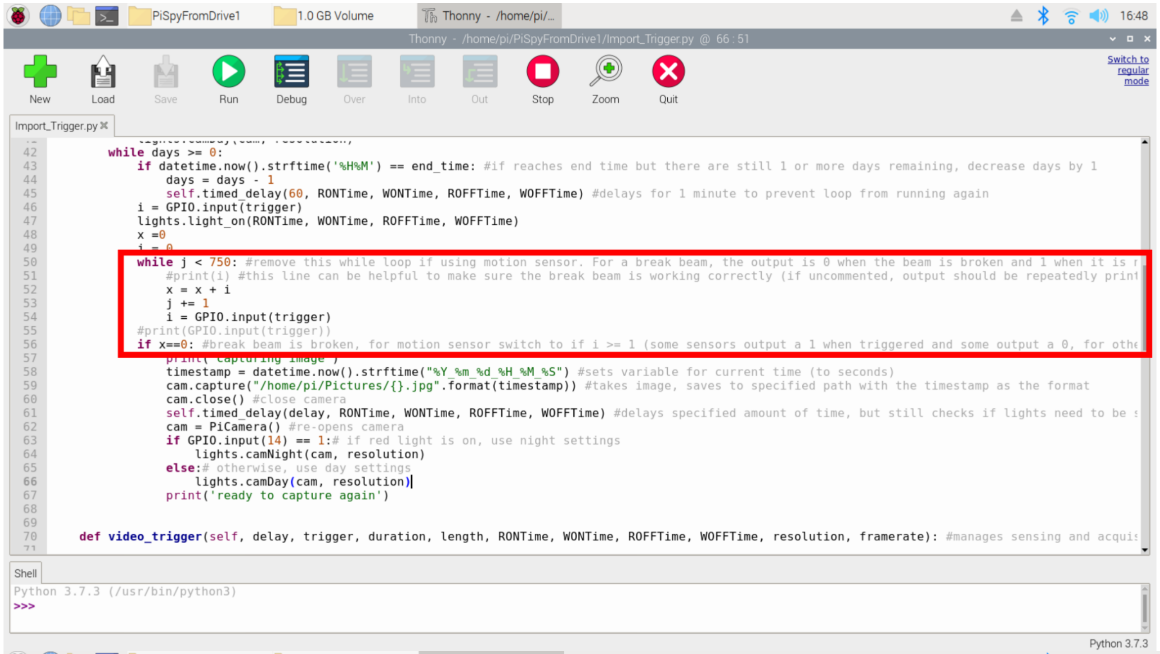

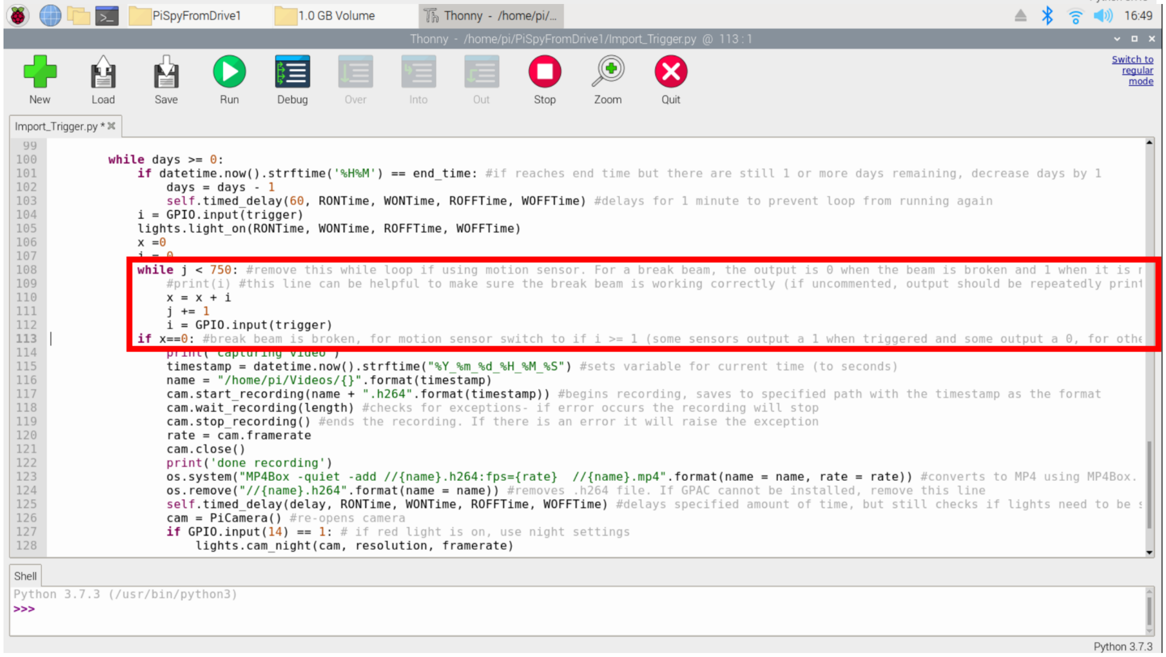

Using input trigger mode

In the PiSpy control window, specify which GPIO pin the trigger source is connected to, using the BCM format (the white text in the above diagram). Additionally, specify in the mm:ss format how long a delay is desired between image or video captures. Note that many sensors (i.e. PIR motion sensors) also have a mechanically adjusted delay setting, so this can also be adjusted to set the device's delay. The PiSpy will use whichever delay is longer. In the image below, 1640x1232 images will be recorded when triggered by a sensor plugged into GPIO 4 with a 15 second delay. Recording will occur for 6 hours.

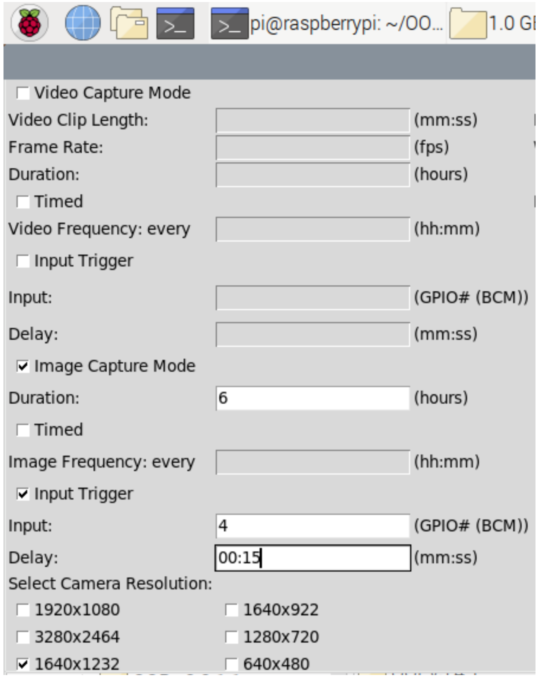

Setting the time on a Raspberry Pi

There are two ways to set the time on a Raspberry Pi. If the computer is connected to the internet and its time zone is set (which is done when the pi is first booted up, or can be done in the localisation tab of the Raspberry Pi Configuration window), the time will set automatically.

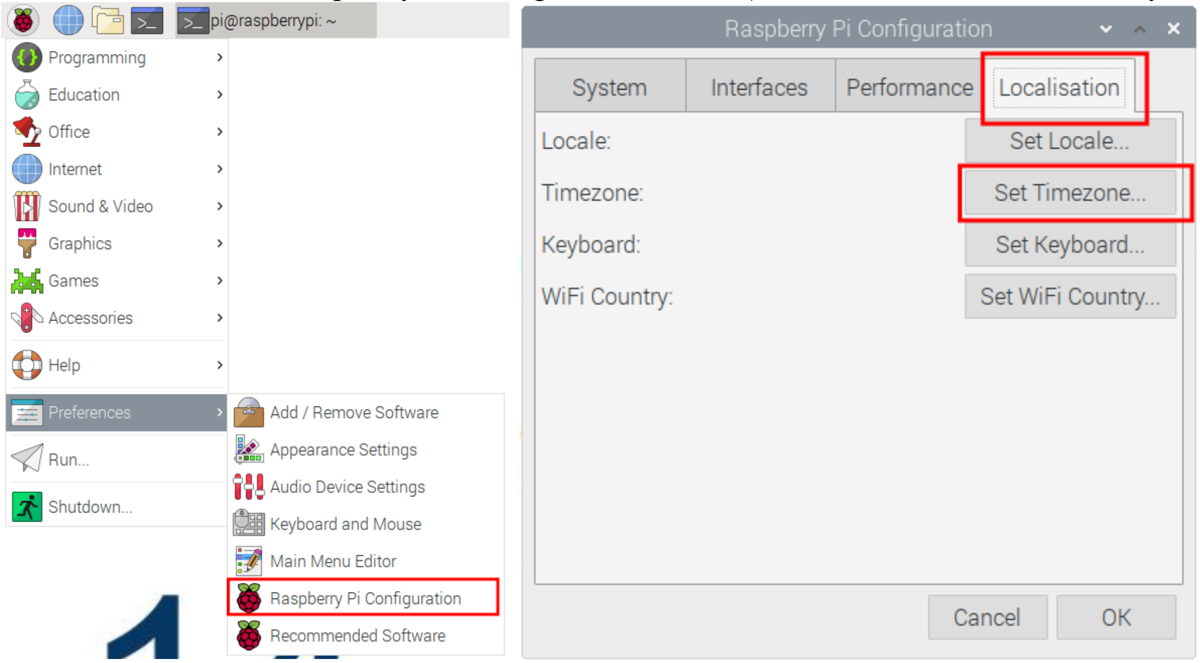

To set the time manually, open a terminal window and type the command (replacing the specifics with whatever the current date/time is):

```
sudo date -s "Tue Jun 15 22:10:11 UTC 2021"
```

Optional: connecting a Real-Time Clock (RTC) to the PiSpy

If, for connectivity, security, or other reasons it will not be possible to connect your PiSpy to the internet, a physical RTC can be connected to the GPIO pins to maintain accurate timing on the device. There are several different RTC options available that we have tested and confirmed to work with the PiSpy, but note that some of these use the same GPIO pins that other optional PiSpy add-ons (such as lights or motion sensors) require. Adafruit has a thorough and easy-to-follow tutorial for connecting different RTCs to a Raspberry Pi, and is also a good source to purchase these devices if they are required for your project: https://learn.adafruit.com/adding-a-real-time-clock-to-raspberry-pi.

Adjusting Default PiSpy Settings

By default, the PiSpy camera will have an ISO of 200 during day recording (no lights on or white lights on), and an ISO of 800 during night recording (red lights on). At night, brightness (set to 43 on a scale from 1 to 100) and contrast (set to -8 on a scale from -100 to 100) are also lowered, as this helped improve image quality with the red LED lights on. If the user wants to adjust any of these settings, open the Day_Night_Mode.py file in the PiSpy folder. Then, make the desired adjustments under the camDay function (to change day settings) or the camNight function (to change night settings). Note that there are two camDay and two camNight functions (one for when framerate is specified and one for when it is not. To ensure a change in settings will always be implemented, make sure to adjust settings in both of these functions. In the screenshot below, the functions highlighted in red control day settings, and the ones in blue control night settings. Note, however, that image quality, especially at night, is also effected by the physical setup of the Pi. See *Note on Red Lights at Night* above for slightly more detail on this.

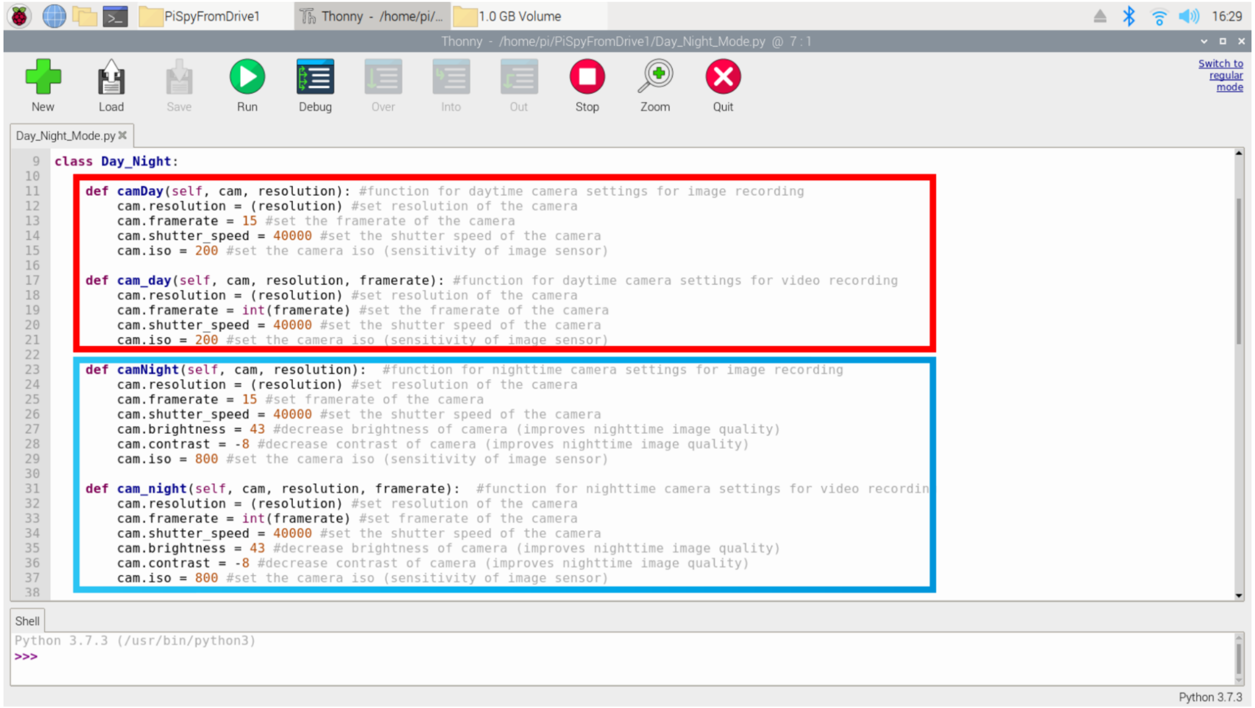

By default, the PiSpy will title images and videos with the time they were recorded (in the format year_month_day_hour:minute), and will store them to the default Pictures folder (/home/pi/Pictures).

To change either of these settings for image recording, open the "Image.py" file. To change the location the images are stored at, replace /home/pi/Pictures in line 20 with the path to the desired folder (make sure to leave the /{}.jpg after the path, as that is what specifies the file name). To change the file name, add any desired text before or after the {} in line 20 (the {} is a placeholder that tells Python where to put the timestamp in the filename). The format of the timestamp itself can be adjusted in line 18.

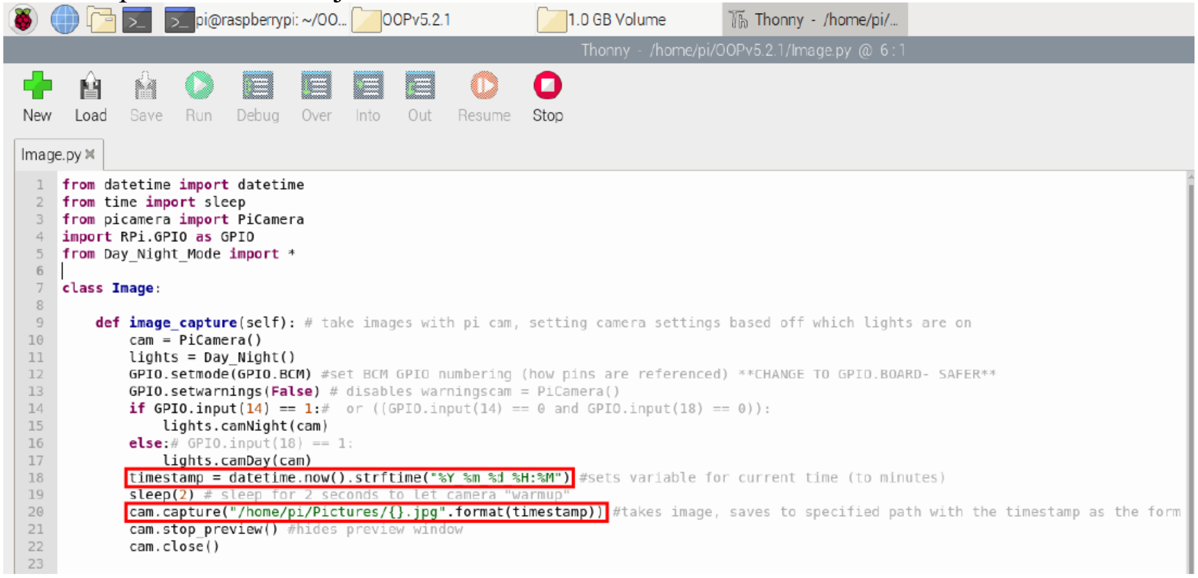

For video recording, open the "Record.py" file. The same steps as above can be used to adjust the file's name and location, except the location/name is determined in line 24, and the format of the timestamp is determined in line 20.

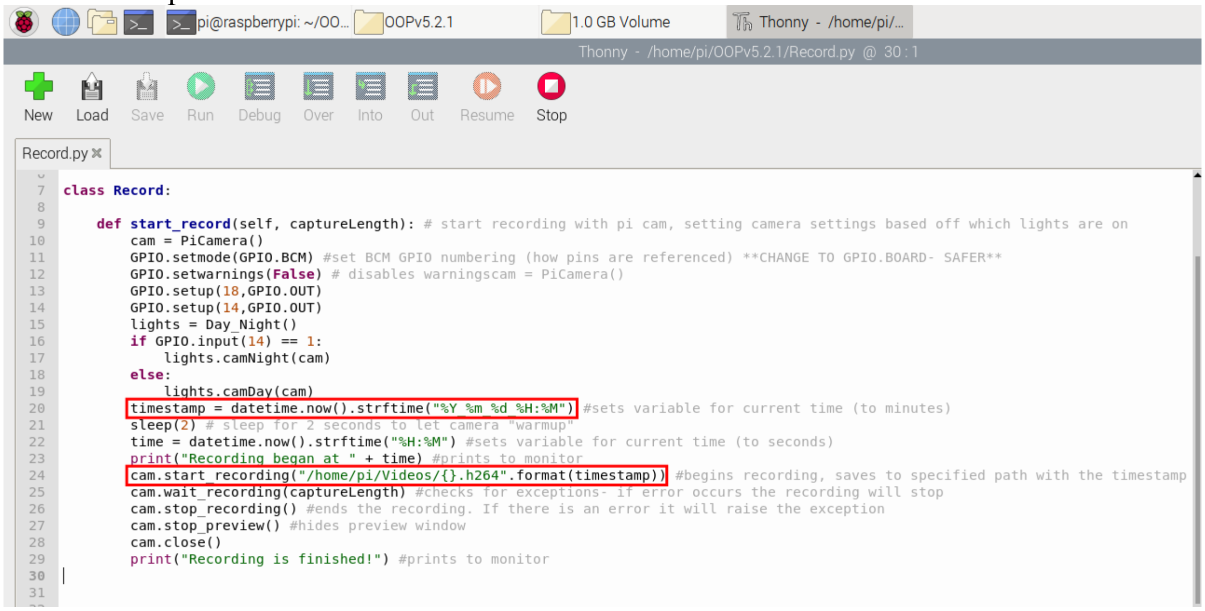

By default, PiSpy uses the a bitrate of 6000000 (6 mb per second), as we found in our testing that this provided sufficient video quality while keeping file sizes small and minimizing frame skipping in the videos. To change this to a different value, update the value specified in line 44 of “Record.py.”

Day_Night_Mode.py | Image.py | Import_Trigger.py | PiSpy.py | Record.py | Time_Lists.py

```
25 class Record:
26
27     def start_record(self, ID, captureLength, resolution, framerate): #captures video, using settings based off which lights are on
28         cam = PiCamera()
29         GPIO.setmode(GPIO.BCM) #set BCM GPIO numbering (how pins are referenced)
30         GPIO.setwarnings(False) # disables warnings
31         GPIO.setup(18,GPIO.OUT) #tells computer that GPIO pins used for red/white lights are outputs
32         GPIO.setup(14,GPIO.OUT) #tells computer that GPIO pins used for red/white lights are outputs
33         lights = Day_Night() #initiates lights class
34         if GPIO.input(14) == 1: #if red lights are on, use night settings
35             lights.cam_night(cam, resolution, framerate)
36         else: #otherwise, use day settings
37             lights.cam_day(cam, resolution, framerate)
38         timestamp = datetime.now().strftime("%Y_%m_%d_%H_%M_%S") #sets variable for current time (to minutes)
39         timestamp = ID + timestamp
40         sleep(2) # sleep for 2 seconds to let camera "warmup"
41         time = datetime.now().strftime("%H:%M:%S") #sets variable for current time (to seconds)
42         print("Recording began at " + time) #prints to monitor
43         name = "/home/pi/Videos/{}".format(timestamp)
44         cam.start_recording(name + ".h264", bitrate = 6000000) #begins recording, saves to specified path with the timestamp as the format
45         cam.wait_recording(captureLength) #checks for exceptions- if error occurs the recording will stop, otherwise records for specified
46         cam.stop_recording() #ends the recording. If there is an error it will raise the exception
47         time = datetime.now().strftime("%H:%M:%S") #sets variable for current time (to seconds)
48         cam.stop_preview() #hides preview window
49         rate = cam.framerate
50         cam.close()
51         os.system("MP4Box -quiet -add //{name}.h264:fps={rate}  //{name}.mp4".format(name = name, rate = rate)) #converts to MP4 using MP4
```

LED PCB Lighting

Designs for white/red LED Printed Circuit Boards (PCBs) are on the PiSpy GitHub repository and can be used to purchase PCBs from fabrication businesses. One or more LED PCBs must be connected to the GPIO board on the Raspberry Pi for lighting control. When the Pi is off, connect the GPIO pins to the corresponding pins on the LED PCB(s) as shown in the diagram below. For GPIO positions, please refer to the pinout diagram above. Alternatively, the pinout command in terminal will display a diagram of the GPIO for your Raspberry Pi model.

If using other lighting outputs, please note the GPIO pins of interest and that the GUI code may need to be modified.

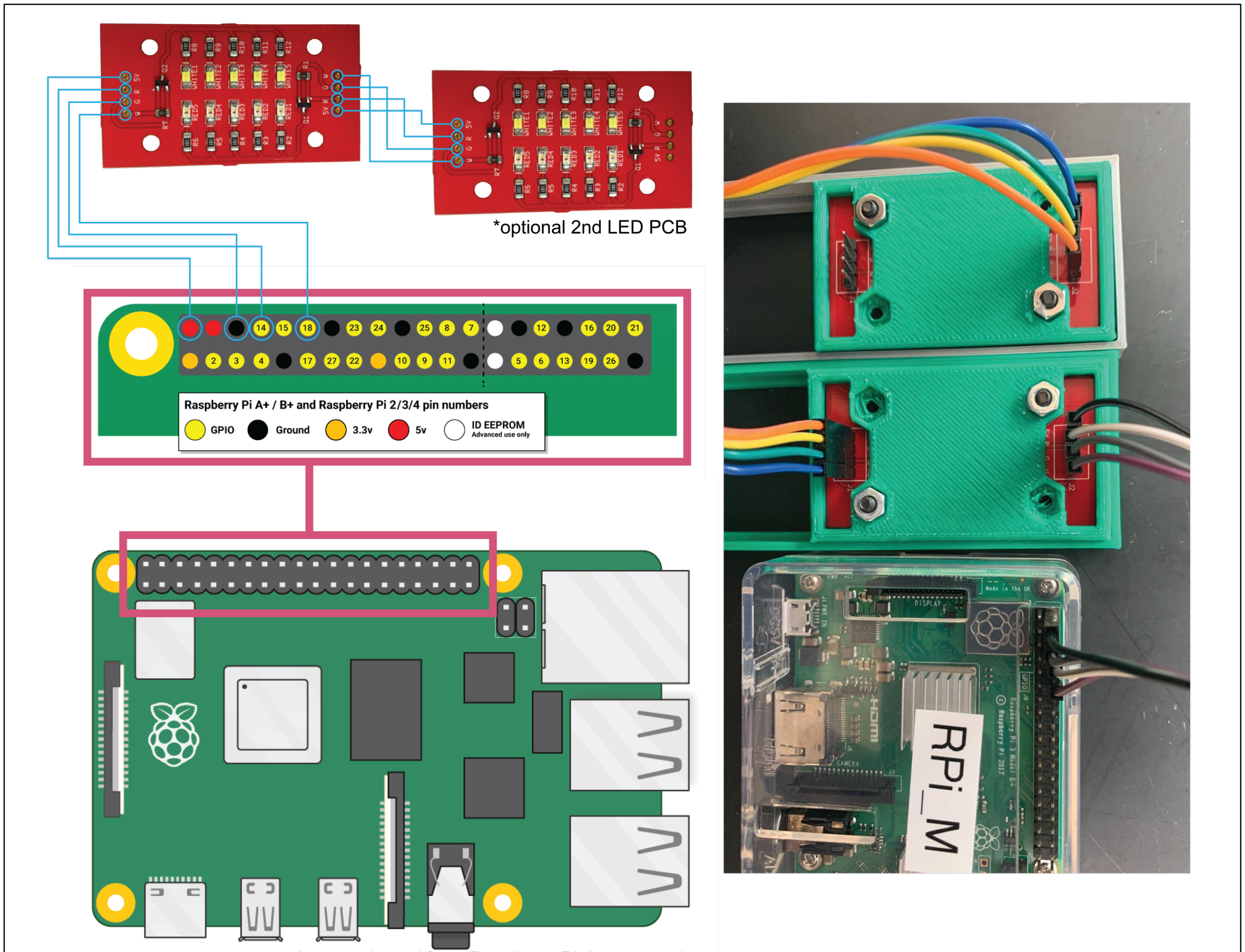

image adapted from Raspberry Pi documentation
